# Inhibitory effect of *trans*-tiliroside on very low-density lipoprotein secretion in HepG2 cells and mouse liver

**DOI:** 10.1007/s11418-023-01756-0

**Published:** 2023-11-16

**Authors:** Akifumi Nagatomo, Mamiko Kohno, Hirosato Kawakami, Yoshiaki Manse, Toshio Morikawa

**Affiliations:** 1 Pharmaceutical Research and Technology Institute, Kindai University, 3-4-1 Kowakae, Higashi-osaka, Osaka 577-8502 Japan; 2 Morishita Jintan Co., Ltd., 11-1 Tsudayamate 2-Chome, Hirakata, Osaka 573-0128 Japan; 3 Antiaging Center, Kindai University, 3-4-1 Kowakae, Higashi-osaka, Osaka 577-8502 Japan

**Keywords:** *Trans*-tiliroside, Rose hi﻿p, Cholesterol, VLDL, Dyslipidemia, Apoprotein

## Abstract

An acylated flavonol glycoside, *trans*-tiliroside (**1**), is found in certain parts of different herbs, including the seeds of *Rosa canina* (Rosaceae)*.* Previous studies on compound **1** have focused on triglyceride (TG) metabolism, including its anti-obesity and intracellular TG reduction effects. In the present study, the effects of compound **1** on cholesterol (CHO) metabolism were investigated using human hepatocellular carcinoma-derived HepG2 cells and mice. Compound **1** decreased CHO secretion in HepG2 cells, which was enhanced by mevalonate in a concentration-dependent manner and decreased the secretion of apoprotein B (apoB)-100, a marker of very low-density lipoprotein (VLDL). Compound **1** also inhibited the activity of microsomal triglyceride transfer proteins, which mediate VLDL formation from cholesterol and triglycerides in the liver. In vivo, compound **1** inhibited the accumulation of Triton WR-1339-induced TG in the blood of fasted mice and maintained low levels of apoB-100. These results suggest that compound **1** inhibits the secretion of CHO as VLDL from the liver and has the potential for use for the prevention of dyslipidemia.

**Graphical abstract:**

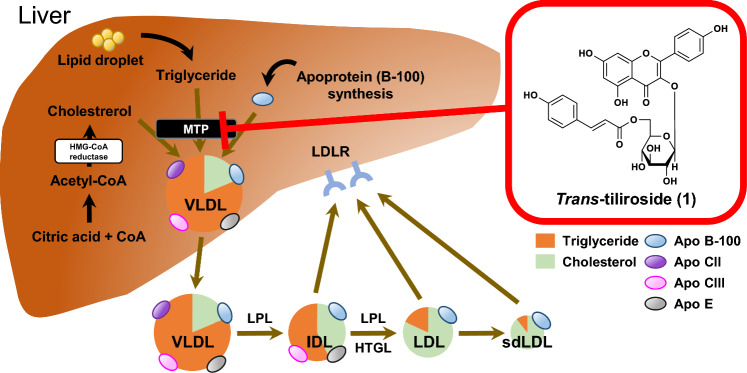

**Supplementary Information:**

The online version contains supplementary material available at 10.1007/s11418-023-01756-0.

## Introduction

Dyslipidemia is a pathological condition in which blood levels of cholesterol (CHO), triglycerides (TG), and lipoproteins are elevated. It is an important risk factor for atherosclerotic cardiovascular disease (ASCVD), which is the most common cause of death not only in Japan but globally [1]. Low-density lipoprotein cholesterol (LDL-C), also known as the "bad cholesterol", plays an important role in transporting CHO and TG to the peripheral tissues. However, excess LDL-C activates the vascular endothelial cells and triggers inflammatory responses in macrophages [2]. These macrophages take up LDL, especially small dense LDL (sdLDL), and transform into foam cells, causing atherosclerosis [3]. Owing to this, reducing LDL-C levels is of primary importance for the prevention of ASCVD.

In circulating blood, CHO and TG form lipoprotein particles together with hydrophilic phospholipids and apoproteins. Apoproteins act as ligands for receptors expressed in various tissues and regulate the metabolic enzymes of lipoproteins [3]. Dietary CHO and TG absorbed from the small intestine are transported as chylomicrons by apoproteins, such as apoB-48, C-II, and E [3]. On the other hand, CHO and TG synthesized in the liver are secreted from the liver to blood as very low-density lipoprotein (VLDL) equipped with apoB-100 and E. VLDL is degraded by lipoprotein lipase (LPL) and converted to LDL via intermediate-density lipoprotein (IDL) [3]. Normally, VLDL is secreted as large VLDL1, composed mostly of TG, which is converted to small VLDL2 by LPL in the blood [4]. Normal LDL is produced from VLDL2, which is taken up via LDL receptors (LDLR) and metabolized in the liver. However, the increased influx of free fatty acids into the liver, accelerated fat synthesis, and suppressed degradation of apoB caused by insulin resistance (IR) results in increased selective production of VLDL1 [4]. As IR attenuates LPL activity, the conversion of VLDL1 to VLDL2 is also reduced, promoting sdLDL formation [4]. sdLDL appears to be more atherosclerosis-inducing because of its long residence time in the circulation, owing to its low affinity for the LDL receptor [5], susceptibility to oxidative modification [6], and tendency to permeate the vessel walls because of its small size [7]. Therefore, it has been reported that the number of sdLDL particles is a more accurate prediction of ASCVD risk than LDL-C [3, 8–10]. In addition, each lipoprotein particle carries one apoprotein molecule, and the amount of apoprotein reflects the number of lipoprotein particles [7]. Therefore, in addition to the level of LDL-C in the blood, the number of these particles can be determined by monitoring the concentration of apoB, which is carried by the LDLs.

**Fig. 1:**
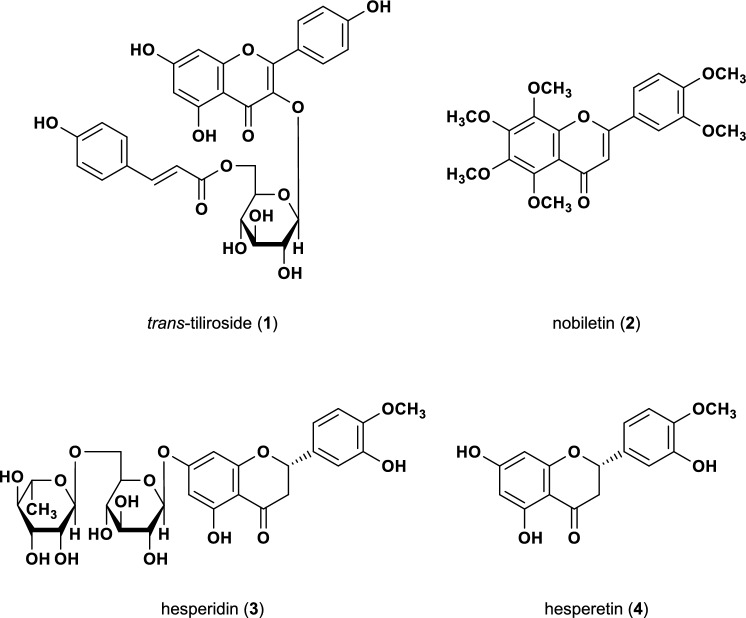
Structures of *trans*-tiliroside (**1**) and flavonoids (**2**–**4**)

Rose hip is a common name for the fruit of Rosaceae plants. The pericarps, seeds, and seed oil of *Rosa canina* and *R. rubiginosa* [11] are commercially available and used in jams, beverages, vitamin C supplements, cosmetic ingredients, and other products [12, 13]. Rose hip has traditionally been used as folk medicine in Europe for the treatment of rheumatism and osteoarthritis [14]. The acylated flavonol glycoside *trans*-tiliroside (**1**) (Fig. 1) contained in rose hip seeds has been reported to exhibit various pharmacological activities, including anti-inflammatory [15], antioxidant [16], anticancer [17, 18], tyrosinase inhibitory [19], and hepatoprotective [20] effects. We have previously reported that compound **1** potently suppresses body weight gain in mice [21, 22], increases mRNA expression of peroxisome proliferator-activated receptor α [21], which regulates lipid metabolism, and reduces intracellular TG and enhances glucose metabolism in human hepatocellular carcinoma-derived HepG2 cells [22, 23]. Studies on lipid metabolism of compound **1** have focused on the decrease in TG in the liver and muscle [21, 24], with few studies on CHO having been conducted.

In this study, we investigated the effects of compound **1** on the secretion of CHO, TG, and apoB-100 in HepG2 cells and mice. We also evaluated the inhibitory effects of nobiletin (**2**) [25–27], hesperidin (**3**) [28–30], and hesperetin (**4**) [31, 32] (Fig. 1), the citrus-derived components with reported anti-hyperlipidemic effects, on CHO secretion in HepG2 cells and compared their mode of action with that of compound **1**.

## Results

### Effects of *trans*-tiliroside (1) and flavonoids (2–4) on CHO secretion in HepG2 cells

**Fig. 2:**
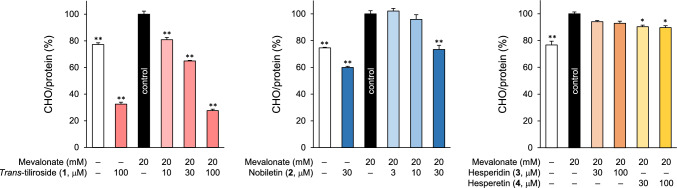
Effects of compounds **1**–**4** on CHO secretion in HepG2 cells. HepG2 cells were treated with **1**–**4** in 20 mM mevalonate-supplemented DMEM (FBS and phenol red-free) for 24 h. The CHO content in the medium was determined using the Amplex^®^ Red assay. Each bar represents the mean ± S.E.M. (*n* = 4). Significantly different from the control, *p < 0.05, **p < 0.01 (Dunnett’s test)

CHO biosynthesis in the liver is regulated by the rate-limiting enzyme, 3-hydroxy-3-methyl-glutaryl-coenzyme A (HMG-CoA) reductase, which catalyzes the synthesis of mevalonate, followed by several steps to produce CHO [33]. Chen et al. reported that the addition of mevalonate to incubation medium increased CHO production in HepG2 cells [34]; we used modified conditions in the present study. The CHO concentration in the medium was standardized based on the protein concentration measured after sonication of the cells. We observed that the addition of mevalonate to serum-free DMEM for 24 h significantly increased the amount of CHO secreted into the medium in HepG2 cells in a concentration-dependent manner, while cell viability decreased at higher concentration of mevalonate (Table S1). Thus, the concentration of mevalonate was optimized to 20 mM. As shown in Fig. 2 and Table S2, the addition of 20 mM mevalonate to serum-free DMEM for 24 h increased the amount of CHO secreted into the medium by approximately 1.3-fold. Compared to the addition of mevalonate alone, compound **1** and **2** significantly reduced the CHO concentration at 100 μM (80.8 ± 1.7% at 10 μM, 64.9 ± 0.5% at 30 μM, 27.7 ± 1.0% at 100 μM) and 30 μM (73.4 ± 2.3%), respectively. Interestingly, 100 μM of compound **1** reduced CHO production to approximately 40% of its basal level, regardless of the presence of mevalonate. Compound **3** showed no effect on CHO secretion, and compound **4** had only a slight inhibitory effect on CHO secretion (90.4 ± 2.2% at 30 μM, 89.6 ± 1.5% at 100 μM).

### Effects of *trans*-tiliroside (1) and nobiletin (2) on HepG2 cell viability

**Fig. 3:**
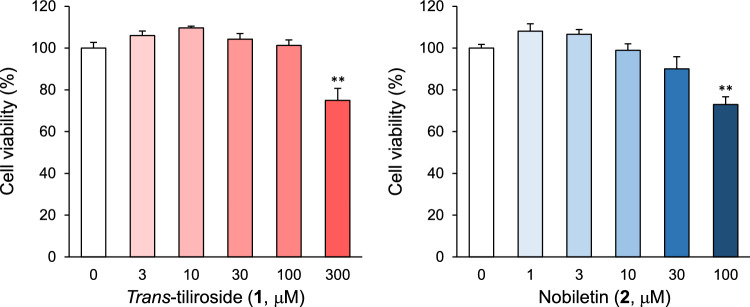
Effects of compounds **1** and **2** on HepG2 cell viability. HepG2 cells were treated with various concentrations of **1** or **2** for 24 h, and cell viability was measured using the WST-8 assay. Each bar represents the mean ± S.E.M. (*n* = 4). Significant difference from the control, **p < 0.01 (Dunnett’s test)

Next, the optimal concentrations of compounds **1** and **2** were determined. The WST-8 assay was used to determine the upper concentration limit at which compounds **1** and **2** did not affect the cells. HepG2 cells were cultured in DMEM supplemented with compounds **1** or **2** for 24 h, and the amount of soluble formazan produced 2 h after the addition of WST-8 was evaluated by measuring the absorbance at 450 nm. As shown in Fig. 3 and Table S3, absorbance significantly decreased to 74.9 ± 5.8% and 72.9 ± 3.7% at 300 μM and 100 μM, respectively, for compounds **1** and **2**. Based on these results, the maximum concentration of compounds **1** and **2** was set at 100 μM and 30 μM, respectively, in subsequent experiments.

### Effects of *trans*-tiliroside (1) and nobiletin (2) on apoB-100 secretion in HepG2 cells

**Fig. 4:**
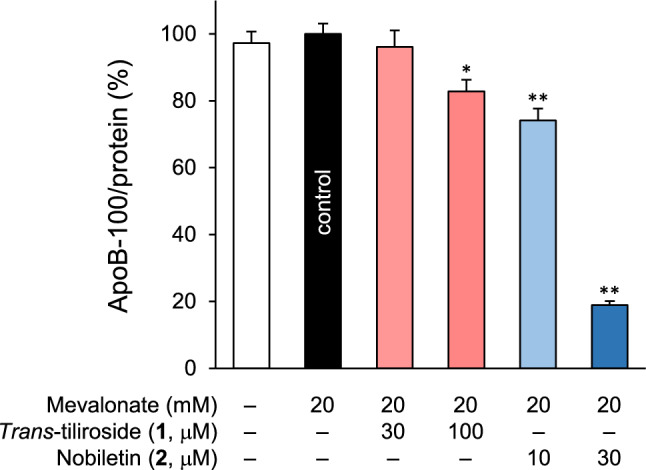
Effects of compounds **1** and **2** on apoB-100 secretion in HepG2 cells. HepG2 cells were treated with **1** or **2** in 20 mM mevalonate-supplemented DMEM (FBS-and phenol red-free) for 24 h. The apoB-100 content in medium were determined by ELISA using commercial kit. Each bar represents the mean ± S.E.M. (*n* = 4). Significantly different from the control, *p < 0.05, **p < 0.01 (Dunnett’s test)

For apoB-100 production, an indicator of VLDL particle count, the addition of mevalonate to the medium slightly increased its level but this was not statistically significant (Fig. 4 and Table S4). Compounds **1** and **2** showed a concentration-dependent inhibition of apoB-100 secretion compared with the addition of mevalonate alone. In particular, compound **2** showed a significant reduction, with apoB-100 production at 30 μM being 18.9 ± 1.2% that of mevalonate alone.

### Effects of *trans*-tiliroside (1) and nobiletin (2) on MTP activity

MTP, a microsomal triglyceride transfer protein, plays an important role in the secretion and assembly of lipoprotein-containing apoB in the liver and intestines [35]. MTP inhibition leads to hypolipidemic activity by preventing the secretion of apoB-containing lipoproteins [36]. Herein, MTP activity was measured as the change in fluorescence intensity due to TG transfer from donor particles containing fluorescently labeled TG in a self-quenched state to acceptor particles. Therefore, the fluorescence intensity increased as the TG was transferred. First, we examined whether each sample had a direct effect on HepG2-derived MTP (Table 1). The results revealed that compound **1** strongly inhibited MTP activity in a concentration-dependent manner and **2** had no effect on MTP activity. Table 1 also indicates the results of the evaluating MTP activity from mevalonate and sample-treated HepG2 cells. MTP activity was slightly increased in cells cultured in medium containing mevalonate. In contrast, MTP activity was not altered by pretreatment with compound **1**. Interestingly, compound **2**, which inhibits CHO and apoB-100 secretion from HepG2 cells as previously stated, accelerated MTP activity after 24 h of treatment.

**Table 1:** Effect of compounds **1** and **2** on MTP activity

Treatment	Concentration (µM)	Mevalonate (20 mM)	Increase of fluorescence intensity (× 10^3^, arbitrary unit)			
			0 h	3 h	6 h	24 h
*Direct inhibition*^a^						
Vehicle	‒	‒	0.0 ± 0.0	23.8 ± 0.5	26.8 ± 0.8	39.5 ± 0.5
*Trans*-tiliroside (**1**)	10	‒	0.0 ± 0.0	22.4 ± 1.1	25.9 ± 0.9	38.8 ± 1.0
	30	‒	0.0 ± 0.0	18.1 ± 0.8**	21.4 ± 0.9**	35.5 ± 0.9**
	100	‒	0.0 ± 0.0	3.3 ± 0.3**	3.9 ± 1.1**	13.7 ± 1.2**
Nobiletin (**2**)	3	‒	0.0 ± 0.0	23.4 ± 0.3	26.1 ± 0.5	38.9 ± 0.7
	10	‒	0.0 ± 0.0	23.4 ± 0.3	26.0 ± 0.5	37.9 ± 0.6
	30	‒	0.0 ± 0.0	23.8 ± 0.3	27.9 ± 0.4	39.3 ± 0.4
*Pretreatment*^b^						
Vehicle	‒	‒	0.0 ± 0.0	9.5 ± 0.3	13.3 ± 0.6	19.5 ± 0.9
Mevalonate	‒	+	0.0 ± 0.0	8.8 ± 0.6	12.6 ± 0.2	22.8 ± 0.9*
*Trans*-tiliroside (**1**)	100	+	0.0 ± 0.0	10.0 ± 0.3	14.3 ± 0.1	22.0 ± 0.8
Nobiletin (**2**)	30	+	0.0 ± 0.0	12.5 ± 0.3**	17.9 ± 0.7**	30.8 ± 0.5**

Each value represents the mean ± S.E.M. (*n* = 4). Significantly different from the vehicle, *p < 0.05, **p < 0.01 (Dunnett’s test)

^a^Crude MTP prepared from untreated HepG2 cells. The samples were then added to the working solution of the MTP activity assay kit

^b^HepG2 cells were pretreated with 100 µM of compound **1**- or 30 µM of compound **2**- containing medium for 24 h, and crude MTP was prepared by homogenizing the cells. The increments of fluorescence intensity from 0 h were calculated

### Effects of *trans*-tiliroside (1) and nobiletin (2) on VLDL secretion in mouse liver

**Fig. 5:**
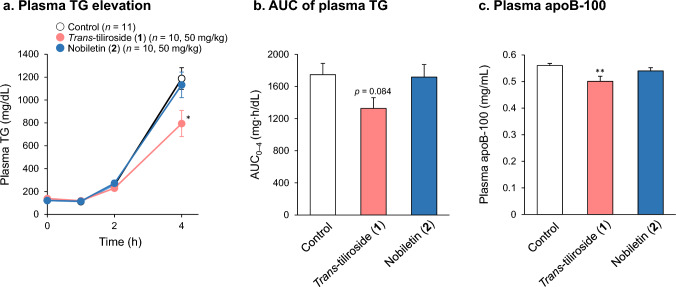
Effects of compounds **1** and **2** on plasma TG and apoB-100 levels in Triton WR-1339-treated mice. The mice were orally administered the sample solution for 7 days. On the sixth day, the mice were deprived of food after sample administration. After 18 h, Triton WR-1339 (400 mg/kg) was injected (i.p.) 1 h after the final administration of the sample. Blood samples were collected at 0, 1, 2, and 4 h after Triton WR-1339 injection. Plasma TG concentration (**a**) was measured calorimetrically, and the area under the curve (AUC_0–4_) (**b**) was calculated using the trapezoidal method. Plasma apoB-100 concentration at 4 h after Triton WR-1339 injection (**c**) was measured using a commercial ELISA kit. Each bar represents the mean ± S.E.M. (*n* = 10–11). Significantly different from the control, * *p* < 0.05, ** *p* < 0.01  (Dunnett’s test)

The most endogenous TGs and CHO secreted from the liver are as VLDL. As mentioned above, VLDL is converted to IDL when TG is degraded by LPL in the circulation. Therefore, the ability of the liver to secrete VLDL can be evaluated by inactivating LPL and measuring the changes in blood TG levels. In this study, we orally administered the test samples to mice for 7 days, followed by intraperitoneal administration of Triton WR-1339, an LPL inhibitor, to halt LPL activity, and measured blood TG levels over time. As a result, the blood TG concentration in the group that received 50 mg/kg of compound **2** was similar to that in the control group, while that in the group that received 50 mg/kg of **1** was significantly lower at 4 h after administration of Triton WR-1339 (Fig. 5a and Table S5). The area under the curve of blood TG concentration (AUC_0–4_) showed a tendency to decrease in the compound **1**-administered group (Fig. 5b and Table S5). Furthermore, the concentration of apoB-100 was lower in the compound **1** group than in the control group (Fig. 5c and Table S6). No effect on the body weight of the mice was observed during the period of sample administration (Table S7).

## Discussion

The currently used drugs for dyslipidemia include HMG-CoA reductase inhibitors, anion exchange resins, CHO transporter inhibitors that inhibit CHO absorption from the small intestine, fibrates that enhance lipid metabolism, polyunsaturated fatty acids such as DHA/EPA, and proprotein convertase subtilisin/kexin type 9 inhibitors [37]. However, the treatment of dyslipidemia begins with lifestyle improvements, such as smoking cessation, reduced alcohol intake, and aerobic exercise, to control LDL-C and TG to appropriate levels. If lipid management is inadequate using the aforementioned strategies. Statins, HMG-CoA reductase inhibitors, are the most frequently used drugs for dyslipidemia. However, they are associated with a high incidence of statin-associated muscle symptoms such as rhabdomyolysis, which may contribute to decreased adherence [38]. Therefore, there is an increasing need for dietary supplements and nutraceuticals (nutraceuticals) as integrative therapy in combination with pharmaceuticals or as adjunctive therapy when LDL-C does not reach target levels with drug therapy [39].

Several food-derived ingredients have been reported to exhibit lipid-lowering effects, including red yeast rice, bergamot, and artichoke leaf extract [40]. In addition, there are several meta-analyses of clinical trials, including single compounds such as flavonoids and dietary fiber, as well as their combinations [40, 41]. Various pharmacological functions have been reported for flavonoids derived from citrus fruits, especially hesperidin (**3**) [28], a combination of hesperetin (**4**) combined with rutinose. Mono-glucosyl hesperidin is a compound in which one molecule of glucose is added to compound **3** to improve its solubility in water. It is used in Japan as a food for specified health uses (FOSHU), with the aim of reducing blood TG levels [42, 43]. However, under our test conditions, both compounds **3** and **4** showed weak effects on CHO secretion from HepG2 cells (Fig. 2).

In the present study, compound **1** inhibited CHO and apoB-100 secretion in HepG2 cells (Figs. 2 and 4). Compound **1** strongly inhibited MTP activity directly in a concentration-dependent manner, and pretreatment with **1** and mevalonate-containing medium showed little effect on MTP activity (Table 1), suggesting that **1** may not affect the expression of MTP. These results suggested that MTP suppression may be one of the mechanisms underlying the inhibitory effects of compound **1** on CHO secretion. Lomitapide, developed as an MTP inhibitor, is approved for the treatment of homozygous familial hypercholesterolemia (hoFH) and strongly reduces LDL-C levels in patients with FH [44, 45]. However, inhibition of VLDL secretion induces nonalcoholic fatty liver disease (NAFLD), and the overall inhibition of MTP may result in fatty liver or liver injury [46]. Selective MTP inhibitors for the small intestine, where the expression of MTP is as high as that in the liver, are also being developed [47, 48]. Lomitapide has also been reported to cause adverse effects on the liver, but in a study using Zucker fatty rats, it was reported to reduce fat accumulation in the liver when used in combination with triiodothyronine, which enhances fatty acid β-oxidation [49]. Compound **1** lowers hepatic fat content in KK-*A*^*y*^ mice [24], a mouse model of type 2 diabetes, and reduces TG accumulation in HepG2 cells [23], suggesting that even if MTP inhibition suppresses VLDL secretion from the liver, it may reduce the risk of developing hepatic steatosis. In addition, the decrease in the concentration of apoB-100 (Fig. 4) and the direct inhibitory effect on MTP (Table 1) were less potent than the decrease in the CHO concentration in the medium (Fig. 2). Therefore, intracellular TG reduction may have a greater effect on VLDL formation. This is supported by the fact that compound **1** inhibited not only the increased CHO production induced by mevalonate but also basal secretion, as shown in Fig. 2.

The effect of compound **1** on VLDL secretion in vivo was evaluated in a mouse model of hyperlipidemia induced by the intraperitoneal administration of Triton WR-1339. Triton WR-1339, a nonionic surfactant, is known to induce hypercholesterolemia [50–53]. Triton WR-1339 inhibits the activity of lipoprotein lipase, causing the accumulation of triglycerides and VLDL in the blood and stimulating HMG-CoA reductase activity, significantly increasing CHO synthesis in the liver [54]. This increase in TG levels is thought to be due to the secretion of VLDL in the liver. Compound **1** inhibited the increase in blood TG and apoB-100 levels after administration of Triton WR-1339 (Fig. 5), suggesting that compound **1** inhibited VLDL secretion from the liver in vivo, similar to the results obtained in HepG2 cells. During the administration of compound **1**, the body weight of the mice did not change from that of the control (Table S7). The decrease in blood TG was not considered to be due to changes in body weight or food intake. Meanwhile, the bioavailability of compound **1** has been reported in a plant extract containing **1**. The maximum plasma concentration (C_max_) of compound **1** was 21.34 ng/mL (approximately 35.9 nM) when **1** was administered at a dose equivalent to 20.4 mg/kg [55]. This concentration in plasma is much lower than the effective concentration of **1** in our study. In addition, the apparent permeability of compound **1** was reported to be low in the small intestinal Caco-2 cell model [56]. However, co-incubation with kaempferol increases transfer of compound **1** from the apical to the basolateral side [56]. On the other hand, Juárez Ramírez et al. reported that compound **1** is metabolized to phloroglucinol and *p*-hydroxyphenyl acetic acid through kaempferol in vivo [57]. The effect of compound **1** on VLDL secretion in this study may result from the enhancement of absorption of **1** by kaempferol produced in the gastrointestinal tract and the cumulated inhibitory effect on VLDL formation in the liver by continuous administration for 7 days. Further studies are needed considering the pharmacokinetics of compound **1**. Other limitations of this study include a lack of experiments on the long-term administration of compound **1** and its effects on CHO-loaded animals. The determination of the preventive effect of compound **1** on dyslipidemia via suppression of VLDL secretion still remains an intriguing question.

Compound **2** is a polymethoxyflavone with multiple hydroxyl groups substituted with methoxyl groups and is a flavonoid unique to citrus fruits. Flavonoids from citrus fruits have been reported to reduce the risk of CVD [58–60], with compound **2** having been shown to reduce total CHO, TG, and LDL levels in the blood [61]. In the present study, compound **2** strongly inhibited the secretion of CHO and apoB-100 in HepG2 cells but had no direct effects on MTP activity (Figs. 2 and 4, and Table 1). On the contrary, compound **2** enhanced MTP activity by pretreatment with **2** and mevalonate-containing medium for 24 h (Table 1). Compound **2** has been reported to enhance expression and activity of the LDLR, and decrease expression and activity of MTP in HepG2 cells [25, 26]. Our results suggest that compound **2** does not directly affect MTP activity in HepG2 cells, but may increase MTP expression under certain conditions. Furthermore, no effect was observed on blood TG elevation or apoB-100 concentration after Triton WR-1339 administration (Fig. 5). In a previous study that used LDLR–deficient mice, compound **2** significantly decreased blood lipid levels and suppressed TG secretion [26]. However, it did not affect the expression of MTP or Acyl-CoA: diacylglycerol acyltransferase, which is involved in the synthesis of TG, with a mechanism of action that mainly decreases the amount of TG in the liver due to increased β-oxidation [26]. This study was based on the long-term administration of compound **2** in a mixed diet. The estimated dose of compound **2** that calculated from its ratio in the diet (0.3%) and the average mouse food intake was 5–10 times higher than the dose in this study (50 mg/kg). Therefore, the reason why we could not confirm the TG secretion inhibitory effect of compound **2** may be explained by the insufficient dosage for induction of its hypolipidemic effect. The low bioavailability of compound **2** may also be another reason. Compound **2** is hardly soluble in water, but evaluation by the Parallel Artificial Membrane Permeation Assay shows high membrane permeability in the pH of 4.0 and 7.0, consistent pH in the gastrointestinal tract [62]. However, in a pharmacokinetic study in rats, the C_max_ was 1.78 µg/mL (approximately 4.4 µM) when 50 mg/kg of compound **2** was administered [62]. Therefore, it is unlikely that 30 µM, the effective concentration in present study, will be reached in vivo.

## Conclusion

In conclusion, *trans*-tiliroside (**1**) reduced the CHO content in the medium after culturing HepG2 cells in a mevalonate-containing medium. The concentration of apoB-100, the apoprotein that is a component of VLDL, along with CHO and TG, was reduced by administration of compound **1**. In addition, compound **1** inhibited MTP activity in HepG2 cells. In vivo, compound **1** lowered blood TG and apoB-100 concentration in Triton WR-1339-induced hyperlipidemic mice. These results suggest that compound **1** inhibits CHO secretion as VLDL in the liver by inhibiting MTP activity and has potential for use for the prevention of dyslipidemia, although further experiments, such as continuous administration studies, should be conducted.

## Materials and methods

### Materials

*Trans*-tiliroside (**1**) was isolated from the seeds of *Rosa canina*. ^1^H- and ^13^C-NMR spectra are available in Supplementary Information (Figures S1 and S2). Nobiletin (**2**), dimethyl sulfoxide (DMSO), and (±)-3-hydroxy-3-methyl-5-pentanolide ((±)-mevalonolactone) were purchased from FUJIFILM Wako Pure Chemical Co. (Osaka, Japan). Hesperidin (**3**) and hesperetin (**4**) were purchased from Funakoshi Co., Ltd. (Tokyo, Japan). Triton WR-1339 was purchased from Sigma-Aldrich Co., LLC. (St. Louis, MO, USA). (±)-Mevalonolactone is described as mevalonate in the text because it is readily converted to mevalonate in water.

### Cell culture

HepG2 cells (RCB1648) purchased from Riken Cell Bank (Tsukuba, Japan) were maintained in Minimum Essential Medium Eagle (MEM, FUJIFILM Wako) containing 10% fetal bovine serum (FBS, Thermo Fisher Scientific Inc., Waltham, MA, USA), 1% MEM nonessential amino acids (FUJIFILM Wako), 100 units/mL penicillin G, and 100 μg/mL streptomycin (FUJIFILM Wako) at 37 °C under a humidified atmosphere containing 5% CO_2_. Each sample was prepared in DMSO to achieve a final concentration of 0.5%.

### Determination on CHO excretion in HepG2 cell containing media

HepG2 cells were seeded into a 48-well tissue culture plate (10^5^ cells/well in 150 μL/well MEM) and cultured for 24 h. The medium was replaced with 150 μL/well of FBS-and phenol red-free DMEM (Sigma-Aldrich) containing mevalonate and a test sample. Twenty-four hours later, the supernatant was collected following determination of CHO concentrations using the Amplex^®^ Red Cholesterol Assay Kit (Thermo Fisher Scientific) according to the manufacturer’s instruction. Briefly, 50 μL of the supernatant was transferred into a 96-well black plate, then 50 μL of the working solution containing 300 μM Amplex^®^ Red reagent, 0.2 U/mL cholesterol esterase, 2 U/mL cholesterol oxidase, and 2 U/mL horseradish peroxidase was added, and incubated for 30 min at 37 °C. Fluorescence intensity was measured using a microplate reader (SH-9000Lab, Hitachi High-Tech Corp., Tokyo, Japan) at excitation and emission wavelengths of 560 and 590 nm, respectively. The cells were homogenized by sonication and the protein concentration was determined using a BCA protein assay kit (FUJIFILM Wako). The CHO content was standardized according to the protein concentration. Aliquots of the collected medium were used to determine apoB-100 levels.

### Cell viability

Cell Counting Kit-8 (WST-8) (Dojindo, Kumamoto, Japan) was used to evaluate cell viability according to the manufacturer’s instructions. Briefly, HepG2 cells were inoculated in a 96-well tissue culture plate (10^4^ cells/well in 150 μL/well in MEM). The cells were cultured until 90% confluence, and the medium was replaced with Dulbecco’s modified Eagle’s medium (DMEM, low glucose, FUJIFILM Wako) containing the test sample. Twenty-four hours later, 5 μL/well of WST-8 was added to each well and incubated for 2 h. The optical density was then measured using a microplate reader (Multiskan Sky, Thermo Fisher Scientific) at a test wavelength of 450 nm. Cell viability was calculated using the following equation:

$${\text{Cell}}\;{\text{viability}}\left( \% \right) = A/B \times 100,$$

where *A* is the absorbance of the test sample and *B* is the absorbance of the control.

### Determination of apoB-100 by ELISA

Determination of apoB-100 in medium or mouse plasma was performed using commercial sandwich ELISA kits and an APOB ELISA Kit (Proteintech Group, Inc., Rosemont, IL, USA) for medium, and a Mouse Apo B ELISA Kit (Abcam plc, Cambridge, UK) for mouse plasma, according to the manufacturer’s instructions. The samples were diluted with the dilution buffer provided in the kit (medium: 3 × ; mouse plasma: 65,000 ×).

### Determination of MTP activity

MTP activity was measured using an MTP Activity Assay Kit (Sigma-Aldrich) according to the manufacturer’s instructions. A protein homogenate containing MTP was prepared from HepG2 cells. The cells were grown in a 75 cm^2^ cultivation flask in MEM until they reached 90% confluence. Twenty-four hours before harvesting, the medium was replaced with fresh DMEM with or without the test sample and mevalonate (20 mM). HepG2 cells were washed thrice with PBS (−) and collected using a cell scraper. After centrifugation, PBS (−) was discarded and cells were suspended in native buffer of the Minute Total Protein Extraction Kit (Invent Biotechnologies, Inc., Plymouth, MN, USA) with protease inhibitors. The cells were disrupted by centrifugation in a shredder column, and the protein concentration in the homogenate was quantified using the BCA method. Cell homogenate (100 μg of protein), sample solution, donor particle, and acceptor particle were mixed in a 96-well black plate and incubated at 25 °C for 24 h. The increase in fluorescence was measured using a microplate reader at excitation and emission wavelengths of 465 and 535 nm, respectively.

### Animals

Male ddY mice were purchased from Kiwa Laboratory Animal Co., Ltd. (Wakayama, Japan). The animals were housed at 23 ± 2 °C and fed a standard chow (MF, Oriental Yeast Co., Ltd., Tokyo, Japan) and supplied filtered tap water. The mice were used 1 week after acclimation. The Kindai University Committee for the Care and Use of Laboratory Animals approved the experimental protocol (KAPR-2023-005).

### Determination of liver triglyceride (TG) secretion in a hyperlipidemic mouse model

Male ddY mice (9 weeks old) were divided into three groups and orally administered the test sample (50 mg/kg) suspended in 5% (w/v) acacia solution for 7 days. The administration volume was 10 mL/kg body weight. On the sixth day of administration, mice were fasted for 18 h. One hour after sample administration on the seventh day, 400 mg/kg of Triton WR-1339, an LPL inhibitor, which was prepared with saline, was administered intraperitoneally. Blood samples were collected into polyethylene tubes (1.5 mL) from the infraorbital venous plexus under isoflurane anesthesia before (0 h) and 1, 2, and 4 h after Triton WR-1339 administration. The blood samples were centrifuged, and plasma TG concentration was determined enzymatically using LabAssay™ Triglyceride (FUJIFILM Wako). Plasma aliquots were used for apoB measurements (vide supra).

### Statistical analysis

Values are expressed as mean ± standard error of the mean (S.E.M.). For statistical analysis, one-way analysis of variance followed by Dunnett’s test was performed using JMP^®^ 9.0.2 (SAS Institute Inc., NC, USA). Statistical significance was set at *p* < 0.05.

### Supplementary Information

Below is the link to the electronic supplementary material.

Supplementary file1 (PDF 620 KB)
