# Supplementary material for: Inhibitory effect of trans-tiliroside on very low-density lipoprotein secretion in HepG2 cells and mouse liver
Source: J Nat Med. 2023 Nov 16;78(1):180–90. doi: 10.1007/s11418-023-01756-0 (PMC10764534; doi:10.1007/s11418-023-01756-0)
Supplement: Supplementary file 1 — Supplementary file1 (PDF 620 KB) [file 11418_2023_1756_MOESM1_ESM.pdf]

Supplementary Information

Inhibitory Effect of *Trans*-tiliroside on Very Low Density Lipoprotein Secretion from HepG2 Cells and Mouse Liver

Akifumi Nagatomo[1,2*] · Mamiko Kohno[2] · Hirosato Kawakami[2] · Yoshiaki Manse[1]
and Toshio Morikawa[1,3*]

[1] Pharmaceutical Research and Technology Institute, Kindai University, 3-4-1 Kowakae, Higashi-osaka, Osaka 577-8502, Japan.

[2] Morishita Jintan Co., Ltd., 11-1 Tsudayamate 2-Chome, Hirakata, Osaka 573-0128, Japan

[3] Antiaging Center, Kindai University, 3-4-1 Kowakae, Higashi-osaka, Osaka 577-8502, Japan.

Table S1. Effects of mevalonate on CHO secretion and cell viability in HepG2 cells S2

Table S2. Effects of *trans*-tiliroside (**1**) and flavonoids (**2**–**4**) on CHO secretion in HepG2 cells........ S2

Table S3. Effects of compounds **1** and **2** on HepG2 cell viability S2

Table S4. Effects of compounds **1** and **2** on apoB-100 secretion in HepG2 cells........ S3

Table S5. Effects of compounds **1** and **2** on plasma TG levels in Triton WR-1339-treated mice S3

Table S6. Plasma apoB-100 concentration at 4 h after Triton WR-1339 injection........ S3

Table S7. Effects of administration of compound **1** and **2** on body weight in mice S3

Figure S1. ^{1}H-NMR (800 MHz, DMSO-d_6) spectrum of compound **1**........ S4

Figure S2. ^{13}C-NMR (200 MHz, DMSO-d_6) spectrum of compound **1**........ S5

Table S1. Effects of mevalonate on CHO secretion and cell viability in HepG2 cells

Treatment	Concentration (mM)	CHO / protein (% of control)	Cell viability (%)
Control	–	100.0 ± 5.0	100.0 ± 0.6
Mevalonate	1	99.2 ± 3.7	108.7 ± 3.7
	5	100.5 ± 4.5	108.5 ± 5.0
	10	118.2 ± 8.3	99.9 ± 4.5
	20	147.8 ± 5.7**	88.9 ± 5.4
	50	119.0 ± 2.9	28.7 ± 0.5**

Each value represents the mean ± S.E.M. ($n = 4$). Significantly different from the control, ** $p < 0.01$ (Dunnett's test).

Table S2. Effects of *trans*-tiliroside (**1**) and flavonoids (**2**–**4**) on CHO secretion in HepG2 cells

Treatment	Concentration (μM)	Mevalonate (20 mM)	CHO / protein (% of control)	Protein concentration (% of control)
Normal	–	–	77.2 ± 1.4**	103.0 ± 2.5
Control	–	+	100.0 ± 2.3	100.0 ± 1.0
Trans-tiliroside (**1**)	10	+	80.8 ± 1.7**	105.1 ± 2.1
	30	+	64.9 ± 0.5**	107.3 ± 0.9*
	100	+	27.7 ± 1.0**	110.6 ± 1.7**
	100	–	32.4 ± 1.4**	109.9 ± 1.5**
Normal	–	–	74.6 ± 0.3**	106.0 ± 0.3
Control	–	+	100.0 ± 1.8	100.0 ± 1.4
Nobiletin (**2**)	3	+	102.1 ± 1.5	101.9 ± 1.0
	10	+	95.9 ± 2.6	103.9 ± 1.6
	30	+	73.4 ± 2.3**	104.7 ± 3.6
	30	–	60.0 ± 0.6**	110.7 ± 0.5**
Normal	–	–	76.7 ± 1.8**	109.2 ± 3.5*
Control	–	+	100.0 ± 1.3	100.0 ± 1.3
Hesperidin (**3**)	30	+	94.1 ± 1.4	103.2 ± 0.9
	100	+	92.9 ± 3.5	102.6 ± 2.2
Hesperetin (**4**)	30	+	90.4 ± 2.2*	112.4 ± 2.1**
	100	+	89.6 ± 1.5**	109.7 ± 2.1**

Each value represents the mean ± S.E.M. ($n = 4$). Significantly different from the control, * $p < 0.05$, ** $p < 0.01$ (Dunnett's test).

Table S3. Effects of compounds **1** and **2** on HepG2 cell viability

Treatment	Cell viability (%)						
	Vehicle	1 μM	3 μM	10 μM	30 μM	100 μM	300 μM
Trans-tiliroside (**1**)	100.0 ± 2.7	–	106.0 ± 2.2	109.6 ± 0.9	104.2 ± 2.7	101.2 ± 2.7	74.9 ± 5.8**
Nobiletin (**2**)	100.0 ± 1.7	108.1 ± 3.6	106.6 ± 2.3	98.9 ± 3.1	90.0 ± 5.9	72.9 ± 3.7**	–

Each value represents the mean ± S.E.M. ($n = 4$). Significantly different from the vehicle, ** $p < 0.01$ (Dunnett's test).

Table S4. Effects of compounds **1** and **2** on apoB-100 secretion in HepG2 cells

Treatment	Concentration (μM)	Mevalonate (20 mM)	ApoB-100 / protein (% of control)
Normal	–	–	97.3 ± 3.4
Control	–	+	100.0 ± 3.1
Trans-tiliroside (**1**)	30	+	96.1 ± 4.9
	100	+	82.9 ± 3.4*
Nobiletin (**2**)	10	+	74.2 ± 3.5**
	30	+	18.9 ± 1.2**

Each value represents the mean ± S.E.M. ($n = 4$). Significantly different from the control, *$p < 0.05$, **$p < 0.01$ (Dunnett's test).

Table S5. Effects of compounds **1** and **2** on plasma TG levels in Triton WR-1339-treated mice

Treatment	Dose (mg/kg)	Plasma TG (mg/dL)				$AUC_{0\text{–}4}$ (mg·h/dL)
		0 h	1 h	2 h	4 h	
Control	–	123.0 ± 12.6	112.4 ± 8.4	257.2 ± 32.0	1187.7 ± 95.1	1747.5 ± 141.2
Trans-tiliroside (**1**)	50	138.6 ± 7.7	119.5 ± 7.0	229.2 ± 15.9	794.6 ± 114.2*	1327.1 ± 133.4#
Nobiletin (**2**)	50	121.4 ± 8.9	115.4 ± 8.4	272.4 ± 25.8	1132.6 ± 111.7	1717.2 ± 155.6

Each value represents the mean ± S.E.M. (n = 10–11). Significantly different from the control, *$p < 0.05$, # $p = 0.084$ (Dunnett's test).

Table S6. Plasma apoB-100 concentration at 4 h after Triton WR-1339 injection

Treatment	Dose (mg/kg)	Plasma apoB-100 (mg/mL)
Control	–	0.56 ± 0.01
Trans-tiliroside (**1**)	50	0.50 ± 0.02**
Nobiletin (**2**)	50	0.54 ± 0.01

Each value represents the mean ± S.E.M. (n = 10–11). Significantly different from the control, **$p < 0.01$ (Dunnett's test).

Table S7. Effects of administration of compound **1** and **2** on body weight in mice

Treatment	Dose	Body weight (g)						
	(mg/kg)	Day 1	Day 2	Day 3	Day 4	Day 5	Day 6	Day 7
Control	–	43.9 ± 0.2	44.2 ± 0.3	43.5 ± 0.3	43.7 ± 0.4	43.8 ± 0.3	43.9 ± 0.4	39.2 ± 0.3
Trans-tiliroside (**1**)	50	43.6 ± 0.3	44.2 ± 0.2	43.5 ± 0.3	44.1 ± 0.4	44.0 ± 0.4	43.9 ± 0.4	39.5 ± 0.5
Nobiletin (**2**)	50	43.7 ± 0.3	44.3 ± 0.4	43.4 ± 0.4	44.0 ± 0.5	44.3 ± 0.5	44.5 ± 0.5	39.5 ± 0.5

Each value represents the mean ± S.E.M. (n = 10–11). No significantly difference was observed. Mice were fasted for 18 h after sample administration at Day 6.

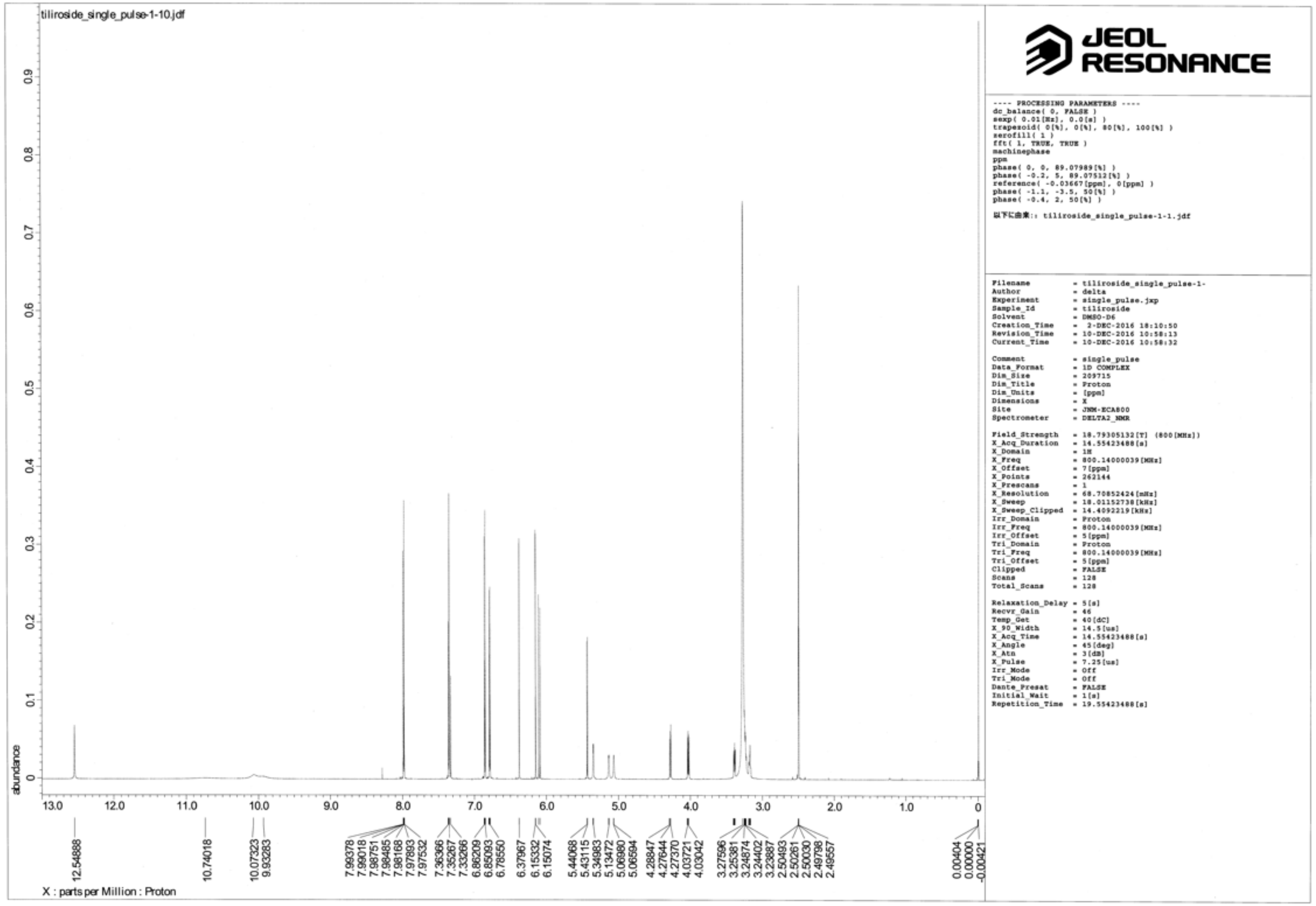

Figure S1. ^{1}H-NMR (800 MHz, DMSO-d_6) spectrum of compound **1**

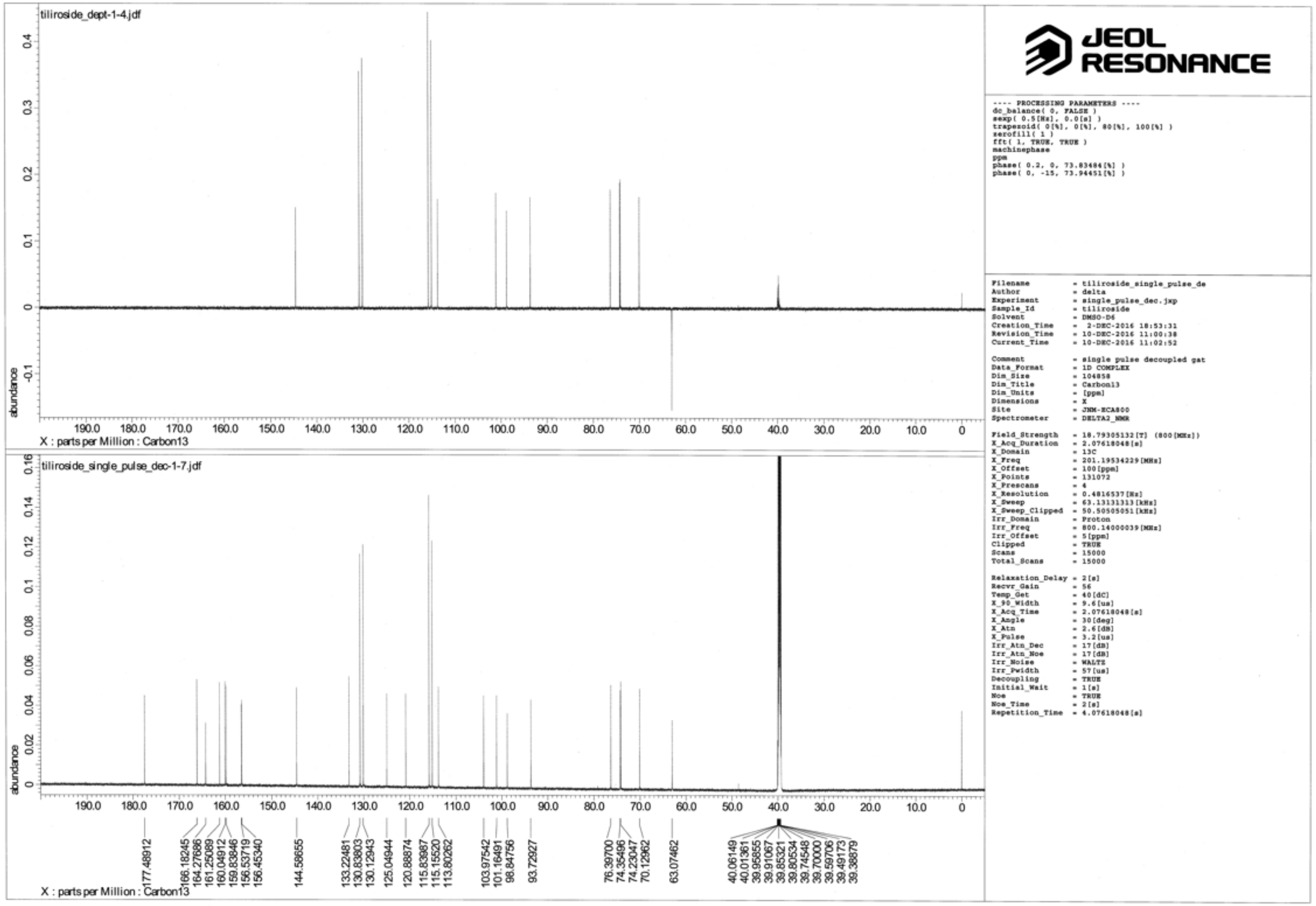

Figure S2. ^{13}C-NMR (200 MHz, DMSO-d_6) spectrum of compound **1**
